# The truncated splice variant of peroxisome proliferator-activated receptor alpha, PPARα-tr, autonomously regulates proliferative and pro-inflammatory genes

**DOI:** 10.1186/s12885-015-1500-x

**Published:** 2015-06-30

**Authors:** Maria Thomas, Christine Bayha, Kathrin Klein, Simon Müller, Thomas S. Weiss, Matthias Schwab, Ulrich M. Zanger

**Affiliations:** 1 Dr. Margarete Fischer-Bosch Institute of Clinical Pharmacology, Auerbachstr. 112, 70736, Stuttgart, and University of Tuebingen, Tuebingen, Germany; 2 University Children Hospital (KUNO), Regensburg University Hospital, Regensburg, Germany; 3 Department of Clinical Pharmacology, University of Tuebingen, Tuebingen, Germany; 4 Present address: MUON-STAT, Klugestraße 28, 70197 Stuttgart, Germany

**Keywords:** Alternative splicing, Fibrates, Hepatocarcinogenesis, PPARA, Primary human hepatocytes, Inflammation, Proliferation, WNT/β-catenin

## Abstract

**Background:**

The peroxisome proliferator-activated receptor alpha (PPARα) controls lipid/energy homeostasis and inflammatory responses. The truncated splice variant PPARα-tr was suggested to exert a dominant negative function despite being unable to bind consensus PPARα DNA response elements.

**Methods:**

The distribution and variability factor of each PPARα variant were assessed in the well-characterized cohort of human liver samples (*N* = 150) on the mRNA and protein levels. Specific siRNA-mediated downregulation of each transcript as well as specific overexpression with subsequent qRT-PCR analysis of downstream genes was used for investigation of specific functional roles of PPARα-wt and PPARα-tr forms in primary human hepatocytes.

**Results:**

Bioinformatic analyses of genome-wide liver expression profiling data suggested a possible role of PPARα-tr in downregulating proliferative and pro-inflammatory genes. Specific gene silencing of both forms in primary human hepatocytes showed that induction of metabolic PPARα-target genes by agonist WY14,643 was prevented by PPARα-wt knock-down but neither prevented nor augmented by PPARα-tr knock-down. WY14,643 treatment did not induce proliferative genes including *MYC*, *CDK1*, and *PCNA*, and knock-down of PPARα-wt had no effect, while PPARα-tr knock-down caused up to 3-fold induction of these genes. Similarly, induction of pro-inflammatory genes *IL1B*, *PTGS2*, and *CCL2* by IL-6 was augmented by knock-down of PPARα-tr but not of PPARα-wt. In contrast to human proliferative genes, orthologous mouse genes were readily inducible by WY14,643 in PPARα-tr non-expressing AML12 mouse hepatocytes. Induction was augmented by overexpression of PPARα-wt and attenuated by overexpression of PPARα-tr. Pro-inflammatory genes including IL-1β, CCL2 and TNFα were induced by WY14,643 in mouse and human cells and both PPARα forms attenuated induction. As potential mechanism of PPARα-tr inhibitory action we suggest crosstalk with WNT/β-catenin pathway. Finally, treatment with WY14,643 in the presence of PPARα-tr resulted in the significant reduction of cell viability of AML12 and human ovarian cancer cell line, SKOV3.

**Conclusions:**

Our data suggest that the truncated PPARα splice variant functions as an endogenous inhibitor of proliferative and pro-inflammatory genes in human cells and that its absence in mouse may explain species-specific differences in fibrate-induced hepatocarcinogenesis.

**Electronic supplementary material:**

The online version of this article (doi:10.1186/s12885-015-1500-x) contains supplementary material, which is available to authorized users.

## Background

The nuclear receptors peroxisome proliferator-activated receptors (PPARs) are ligand-dependent transcription factors involved in diverse physiological roles such as lipid homeostasis, energy metabolism, inflammation, and cellular differentiation and proliferation [42]. The three related PPAR isotypes, PPARα (NR1C1), PPARβ/δ (NR1C2), and PPARγ (NR1C3), share a high degree of homology but differ in tissue distribution and ligand specificity [13]. Because of their central role in regulating energy homeostasis and their often beneficial effects, PPARs are attractive pharmaceutical targets, in particular for the treatment of cardiovascular diseases [40] as well as obesity and other metabolic disorders [8].

A large body of literature described their essential role in cancer [26]. For instance, due to their antiproliferative, proapoptotic, and differentiation-promoting activity, PPARβ/δ and PPARγ agonists have been extensively studied as potential anticancer agents [46]. The role of PPARα in hepatic carcinogenesis appears to be species-dependent. In some rodents, PPARα has been implicated as a key mediator of non-genotoxic hepatocarcinogenesis. Thus, chronic treatment of rats and mice with PPARα agonists (e.g., fibrate drugs) results in increased incidence of liver tumors through a PPARα-mediated mechanism [25, 29]. Importantly, however, these chemicals do not induce cell proliferation in human cells *in vitro* or cancer in humans, suggesting significant differences between human PPARα and rodent Pparα-dependent regulatory pathways [1, 23]. Several factors were suggested to be responsible for the species-specific effects, including differences in the level of receptor expression [24], ligand affinity and other factors involved in PPARα activation [12], as well as the profile of genes induced by mouse Pparα versus human PPARα following treatment with fibrate drugs [22, 44]. Interestingly, *PPARA*-humanized mice essentially lacked susceptibility to the hepatocarcinogenic effects of the peroxisomal proliferator model substance, WY14,643, and other fibrates, suggesting that structural differences between human and mouse PPARα are at least in part responsible for the species difference in hepatocarcinogenesis [22, 36, 44].

**Fig. 1:**
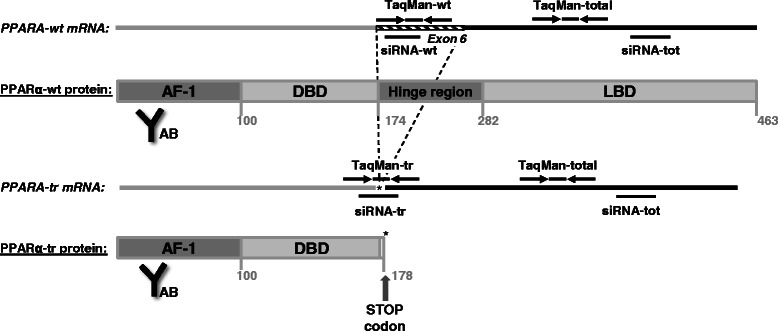
PPARα domain structure and probe locations. PPARα-wild type (wt) and truncated (tr) transcripts are shown as lines with primers and probes for TaqMan gene expression assays indicated schematically above, and siRNA probes below. The generation of an alternative splice variant via introduction of a pre-mature stop codon (asterisk) is shown with the dotted line. The corresponding protein products of two splice isoforms are underlined. The indicated protein domains are: AF-1, activation function 1 domain, DBD, DNA-binding domain, LBD, ligand-binding domain. “AB” indicates the region of antibody binding

One striking discovery, which highlights the significance of sequence differences is the existence of an alternatively spliced transcript variant in humans but not in rodents [9, 24]. The variant human *PPARA*-tr transcript lacks the entire exon 6 due to alternative splicing, leading to a premature stop codon and the generation of a truncated protein (PPARα-tr) with deficient ligand binding domain that is unable to bind to peroxisome proliferator-responsive DNA elements (PPRE) (Fig. 1). Nevertheless, based on luciferase reporter gene assays it has been suggested that PPARα-tr may exert dominant-negative functions [9]. *In vivo* evidence for this hypothesis is entirely lacking for humans. However in jerboas the PPARα-wt/PPARα-tr ratio was shown to depend on the hibernation cycle, thereby affecting the expression of metabolic target genes and lipid storage during feeding and hybernation phases [7]. Whether the endogenous human PPARα-tr has a specific physiological significance in regulating metabolic processes as well as its relevance for hepatocarcinogenesis remained unclear.

Here we used a combination of approaches to investigate the function of PPARα-tr in human and mouse hepatocytes in comparison to the canonical PPARα-wt form. We examined the expression of each *PPARA* form in a cohort of human liver samples on the protein and mRNA levels. Genome-wide correlation analysis with subsequent pathway enrichment analysis indicated a selective role for PPARα-tr as an antiproliferative and anti-inflammatory factor. Experimental manipulation of human and mouse hepatocytes by specific knock-down and overexpression constructs confirmed and further substantiated this hypothesis.

Our data suggest that the truncated PPARα splice variant is differentially regulated and has autonomous functions in human hepatocytes and possibly other cells. Its absence in the mouse may explain species-specific differences in fibrate-induced hepatocarcinogenesis.

## Results

### PPARα-wt and PPARα-tr proteins are differentially regulated in human liver

**Fig. 2:**
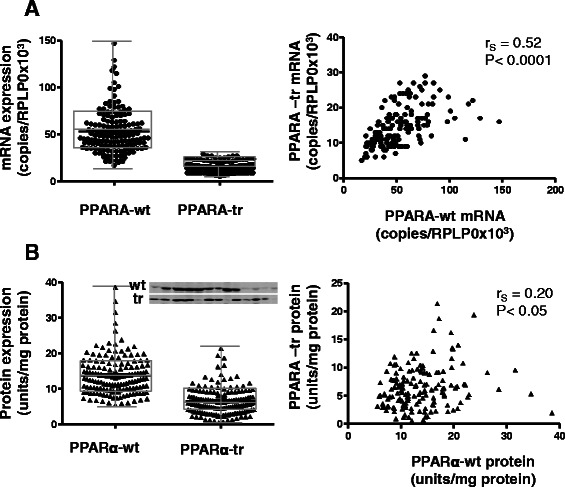
Distribution and correlation of PPARα-wt and PPARα-tr variants in the human liver (*N* = 150). **a** Box-and-whisker plot reflecting the variability of mRNA copies for each PPARA transcript isoform (left-hand side). Expression correlation of both variants on the mRNA level (right-hand side). **b** Box-and-whisker plot reflecting the variability of the absolute amount of protein quantified by western blot for each PPARα isoform (left-hand side) with representative example blot as an insert. Correlation between both variants on the protein level (right-hand side)

We initially hypothesized that levels of endogenous PPARα-tr, given a general dominant negative function, should be negatively related to the expression of PPARα target genes. We therefore assessed the expression of each transcript form in a well-characterized cohort of human liver samples (*N* = 150) [32]. Mean absolute transcript levels of *PPARA-*tr were approximately 5-fold lower compared to *PPARA-*wt (Fig. 2a), in line with previous reports of Gervois et al. [9] and Hanselmann et al. [14]. Both transcript forms varied considerably between the donors (wt, CV = 44 % and tr, 37 %), but their expression was well correlated (Spearman coefficient r_s_ = 0.52; *P* < 0.0001). We used a polyclonal antibody targeting the common N-terminal part of PPARα to simultaneously quantify the full-length (52 kDa) and truncated (30 kDa) protein forms (Fig. 2b). On average, PPARα-tr protein was ~3-fold lower expressed compared to PPARα-wt, indicating either more efficient translation or increased stability of the splice variant compared to the full-length form (Fig. 2b). Both PPARα-wt and PPARα-tr protein levels were not correlated to their respective transcript levels (r_s_ = −0.05, *P* = 0.5, for PPARα-wt and r_s_ = 0.03, *P* = 0.7, for PPARα-tr), indicating significant posttranscriptional regulation. In contrast to the correlated transcript levels, the protein levels of PPARα-wt and PPARα-tr were only marginally correlated to each other (r_s_ = 0.20; *P* < 0.05), suggesting that posttranscriptional regulation mechanisms differ between the two forms. This essential lack of correlation between the two forms resulted in ≈ 30-fold variable PPARα-wt/PPARα-tr ratio in the cohort compared to ≈ 7-fold of PPARα-wt variability, thereby potentially extending the dynamic range of PPARα function. Thus, a putative dominant negative function of PPARα-tr should become apparent by correlating PPARα protein and gene expression levels.

### PPARα-wt and PPARα-tr correlate with different gene sets

**Fig. 3:**
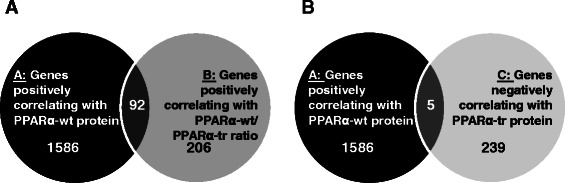
Distribution of the genes using genome-wide correlation analysis in the cohort of human liver samples. **a** Venn diagram demonstrates the intersection (group *I*) between the positively correlated with PPARα-wt (black circle, group A) and with PPARa-wt/PPARa-tr ratio (dark-grey circle, group B) genes following genome-wide correlations between each PPARα protein form with the expression data assessed with Human-WG6v2 Illumina Expression microarrays. **b** Venn diagram shows the overlap between the positively correlated with PPARα-wt (black circle, group A) and with negatively correlated PPARα-tr (light-grey circle, group C) genes following genome-wide correlations between each PPARα protein form with the expression data assessed with Illumina microarrays. The 20 highest-ranked genes of each group are listed in the Additional file 1: Table S1

To test this assumption, we performed Spearman correlation analyses between PPARα protein forms and previously generated genome-wide mRNA expression profiles [32]. A total of 1586 genes were found to be positively correlated to PPARα-wt protein (r_s_ > 0.3, *P* < 0.01, group A, Fig. 3a), compared to only 206 genes that were positively correlated to PPARα-wt/PPARα-tr protein ratio (r_s_ > 0.3, *P* < 0.01, group B, Fig. 3a; see Additional file 1: Table S1 for Top 20 highest-ranked genes). The intersection between the groups A and B comprised only 92 genes (Fig. 3a). Pathway enrichment analyses using Reactome database revealed that genes positively correlated to PPARα-wt belonged to pathways of energy metabolism, specifically in terms of lipid, amino acids and carbohydrate biotransformation, including most of the well-known PPARα target genes (Table 1, Group A). However, these terms were not enriched with either genes positively correlating with PPARα-wt/PPARα-tr ratio nor in the intersection between the two groups (Table 1, Groups B and *Intersection*).

**Table 1:** Pathway enrichment analysis using Reactome database of gene groups defined via correlation analysis of genome-wide gene expression data (for details see Fig. 3)

Selected gene groups	Pathway term	*P*-value	Enrichment score
A: Positively correlating with PPARα-wt protein	Metabolism of lipids and lipoproteins	0.00002	10.4
	Biological oxidations	0.005	2.89
	Integration of energy metabolism	0.011	2.70
	Metabolism of amino acids	0.015	2.48
	Metabolism of carbohydrates	0.017	1.51
B: Positively correlating with PPARα-wt/PPARα-tr protein ratio	Signaling by GPCR	0.07	0.24
	Hormone biosynthesis	0.09	0.17
	Synaptic Transmission	0.19	0.15
	Apoptosis	0.37	0.14
	Integration of energy metabolism	0.40	0.09
Intersection between the groups A and B	Integration of energy metabolism	0.0008	7.79
	Hemostasis	0.002	6.36
	Signaling by BMP	0.08	0.81
	HIV Infection	0.028	0.74
	DNA Repair	0.14	0.55
C: Negatively correlating with PPARα-tr protein	Signaling in Immune system	0.029	1.06
	Cell Cycle, Mitotic	0.047	0.88
	Signaling by Wnt	0.042	0.63
	Apoptosis	0.13	0.60
	IL3 signaling	0.12	0.40
Intersection between the groups A and C	Full name	Functions	
RBMS1	RNA Binding Motif, Single Stranded Interacting Protein 1	Single-stranded DNA binding protein interacting with upstream region of C-MYC gene.	
POFUT1	Protein O-Fucosyltransferase 1	Metabolic relevant enzyme, catalyzer of the fucose attachement to serine/threonine residues.	
DEAF1	DEAF1 Transcription Factor (also: suppressin)	Secreted factor, acts as an inhibitor of cell proliferation by arresting cells in the G0 or G1 phase.	
SLC6A9	Solute Carrier Family 6 (Neurotransmitter Transporter, Glycine), Member 9	Terminates the action of glycine by its high affinity sodium-dependent reuptake	
FLCN	Folliculin	Not clearly classified protein, involved in energy and/or nutrient sensing via AMPK and mTOR signaling pathways.	

Interestingly, within 239 genes negatively correlated to PPARα-tr protein (rs < −0.3, *p* < 0.01; Fig. 3b, Group C), significantly enriched terms included immune system and WNT signalling as well as cell cycle but not classical metabolic pathways regulated by PPARα (Table 1, Group C). Surprisingly, only five overlapping genes were represented in the intersection between the groups A and C. These genes were so far not described as PPARα target genes (Table 1, Intersection between the groups A and C).

Taken together, bioinformatic analysis of the gene groups correlating with either PPARα-wt/PPARα-tr protein ratio as well as of the intersection between PPARα-wt-positive and PPARα-tr-negative correlated genes did not support a significant and general dominant negative effect of PPARα-tr on the function of the canonical receptor. Interestingly, however, the data indicate that PPARα-tr may act rather independently by altering the expression sets of genes different from those regulated by the canonical receptor.

### PPARα-tr targets proliferative and pro-inflammatory genes

**Fig. 4:**
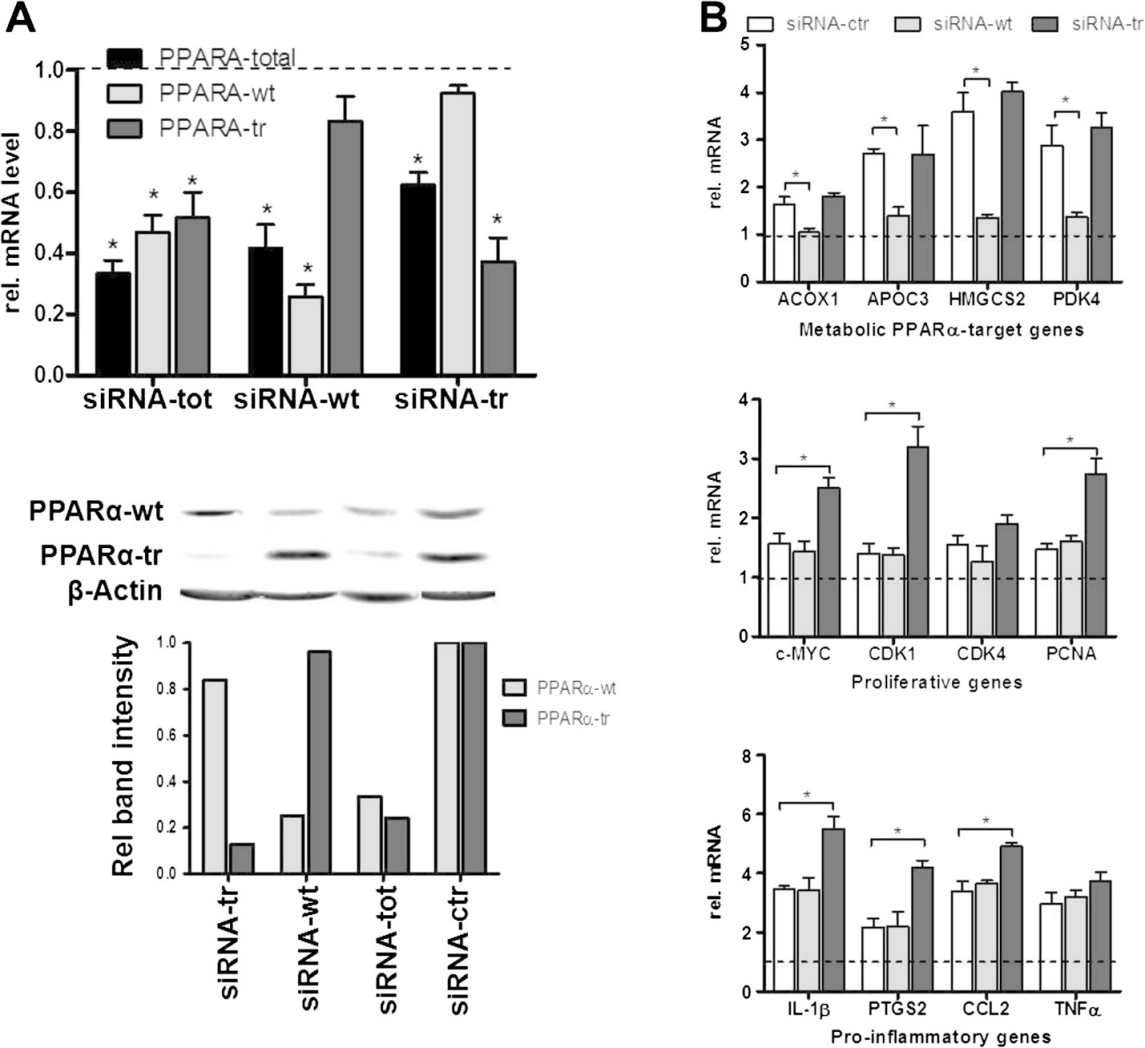
Specific knock-down of PPARα transcript variants in primary human hepatocytes. **a** PHHs (*n* = 3) were transfected with siRNAs targeting PPARA-wt transcript only (siRNA-wt), PPARA-tr only (siRNA-tr), or both transcripts (siRNA-tot). Total and specific mRNA levels were determined by using specific TaqMan assays in comparison to non-targeting siRNA (siRNA-ctr; set to 1 and shown with the dotted line). Results represent means of three PHH donors with two individual replicates. Statistical significance was assessed by paired t-test in comparison to siRNA-ctr. At the bottom, PPARα protein was detected by Western blot analysis in total cell homogenates (50 μg per lane, representative western blot is shown) using a polyclonal antibody targeting the common N-terminal part of PPARα. The immunoreactive bands (upper panel) at 52 kDa (PPARα-wt) and 30 kDa (PPARα-tr) were densitometrically quantified and the intensities shown relative to the siRNA-ctr control. **b** Quantitative RT-PCR analysis of the selected canonical PPARα-target genes was performed in the cell lysates of A) 48 h after the transfection with the indicated siRNAs and treatment with WY14,643, in comparison to the cells transfected with siRNA-ctr and treated with the solvent control, DMSO (the dotted line) (Top panel Quantitative RT-PCR analysis of the selected proliferative genes was performed in the cell lysates of A) 48 h after the transfection with the indicated siRNAs and treatment with WY14,643 (middle panel). Quantitative RT-PCR analysis of the selected pro-inflammatory genes in the cell lysates of A) 48 h after the transfection with the indicated siRNAs and treatment with IL-6, in comparison to the cells transfected with siRNA-ctr and treated with the solvent control, PBS (the dotted line) (bottom panel). * indicates significance *p* < 0.05

To directly determine the effects of each PPARα variant on expression of different classes of target genes in liver cells we designed specific siRNAs (Fig. 1). Selective targeting of each PPARα variant was confirmed in primary human hepatocytes (PHH; Fig. 4a top). In particular, siRNA-tr transfection did not significantly decrease *PPARA*-wt, and siRNA-wt transfection did not decrease *PPARA*-tr. As shown by Western blot analysis, both PPARα protein forms were effectively and specifically downregulated by these siRNAs (Fig. 4a bottom).

The assessment of downstream gene expression effects following application of the specific siRNAs was performed in combination with PPARα activation by WY14,643 in PHH cultures of three independent hepatocyte donors (Fig. 4b). As expected, expression of four selected metabolic PPARα-target genes was significantly induced following treatment with WY14,643 (Fig. 4b, upper panel, white bars). SiRNA-mediated downregulation of *PPARA*-wt resulted in essentially complete block of induction of these genes (light grey bars), while knock-down of *PPARA*-tr did not have an effect (dark grey bars), suggesting that PPARα-tr has neither positive nor negative regulatory functions towards these classical PPARα target genes. The levels of target gene expression following siRNA transfections and treated with the solvent control, DMSO, were used for the normalization and are represented by the dotted line. Additional file 2: Figure S2A (top) shows the mRNA expression changes relative to siRNA-ctr in the absence of the PPARα ligand.

As shown in Fig. 4b (middle panel), the four selected proliferative genes were slightly but not significantly induced by WY14,643 treatment alone, suggesting that they are not directly regulated by PPARα. Consistently, specific knock-down of *PPARA*-wt did not have an effect. However, knock-down of *PPARA*-tr lead to significant, up to 3-fold upregulation of all but one (CDK4) of the four genes. In the Additional file 2: Figure S2A (middle) the mRNA expression changes relative to siRNA-ctr in the absence of WY14,643 are shown.

Four typical pro-inflammatory genes, *IL1B*, *PTGS2*, *CCL2* and *TNF* were measured using a similar set-up with hepatocytes from the same donors as above but challenged with the pro-inflammatory cytokine IL-6 (Fig. 3, bottom panel). The expression levels of all four genes were significantly induced upon 48 hours of IL-6 treatment, demonstrating the triggering of an acute phase response. Except for TNFα, expression was significantly upregulated following selective knock-down of *PPARA*-tr, while *PPARA*-wt had again no effect (Fig. 4, bottom panel). Additional file 2: Figure S2A (bottom) shows the mRNA expression changes relative to siRNA-ctr in the cells treated with the solvent control, PBS.

Taken together, these experiments suggested that endogenous PPARα-tr attenuates the induction of several key proliferative genes by WY14,643 and of key pro-inflammatory genes by IL-6 that are not classical PPARα target genes in primary human hepatocytes.

### Proliferative genes are less sensitive towards PPARα regulation in human versus mouse

Based on the previous experiments we hypothesized that the lack of PPARα-tr in mice could be a key factor for murine fibrate-induced hepatocarcinogenesis. To scrutinize this assumption we used mouse AML12 immortalized hepatocytes. We first verified that AML12 cells do not express PPARα-tr at the transcript and protein level (Additional file 3: Figure S1A and B).

**Fig. 5:**
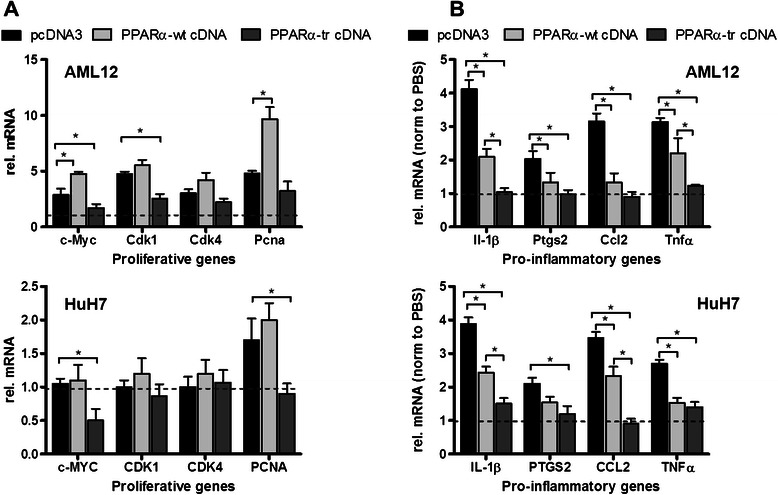
Overexpression of PPARα variants in human and mouse hepatic cell lines. **a** qRT-PCR analysis of the selected proliferative genes following overexpression of each PPARα isoform and treatment with WY14,643 of mouse AML12 (top) and human hepatoma HuH7 cells (bottom). Error bars represent standard deviations of three independent experiments. **b** Quantitative qRT-PCR analysis of the selected pro-inflammatory genes following overexpression of each PPARα isoform and treatment with IL-6 of mouse AML12 (top) and human hepatoma HuH7 cells (bottom). Error bars represent standard deviations of three independent experiments. * indicates significance *p* < 0.05

Treatment of AML12 cells with WY14,643 lead to the induction of all four proliferative genes by ~2.5 to ~5-fold (Fig. 5a top). Transfection of PPARα-wt expression plasmid augmented induction of *Myc* and *Pcna* approximately two-fold. In contrast, transfection of PPARα-tr attenuated induction of *Myc* and *Cdk1* significantly. Less profound, statistically not significant effects were observed for *Cdk4* and *Pcna*. For comparison, exposure of human hepatoma cells HuH7 to WY14,643 induced only *PCNA*. While transfection of PPARα-wt had no augmenting effect on any proliferative gene, PPARα-tr overexpression significantly inhibited *MYC* expression and prevented induction of *PCNA* (Fig. 5a bottom).

In contrast to the proliferative genes, overexpression of both PPARα forms had a significant inhibitory effect on the expression of most pro-inflammatory genes in hepatocytes of both species with PPARα-tr being considerably more effective than PPARα-wt (Fig. 5b).

Taken together these data suggested that these mouse proliferative genes are more susceptible towards PPARα activation than the corresponding human genes but equally or even more effectively inhibitable by PPARα-tr, while both PPARα variants show profound inhibitory effects on the expression of pro-inflammatory genes in both species.

### Inhibitory functions of PPARα-tr can be mediated via WNT/β-catenin pathway and/or via NF-kB pathway

**Fig. 6:**
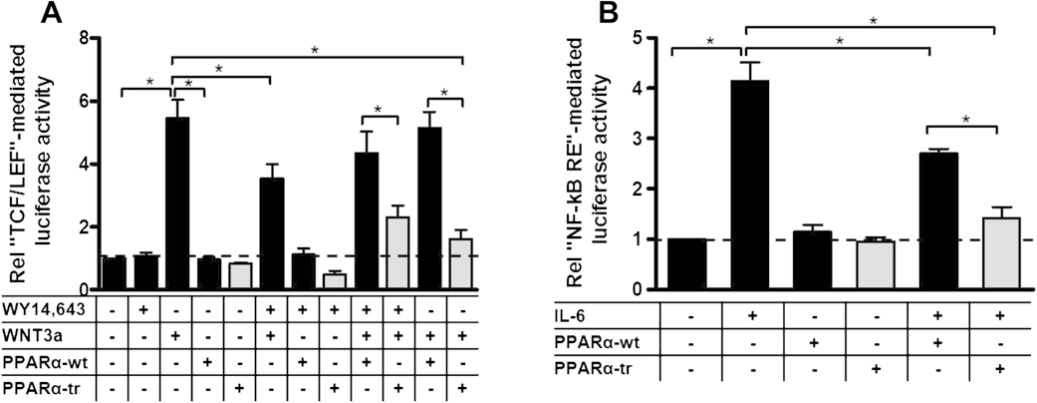
Differential activity of PPARα-wt and PPARα-tr on the WNT/β-catenin and NF-kB promoter binding elements. **a** Quantitative luciferase reporter gene assays with the constructs containing 4xTCF/LEF response elements were performed in human HuH7 cells 48 h after indicated treatments and in total 72 h after the transfection with the indicated constructs. The bars represent the fold induction of luciferase activity normalized to the control state without any treatment (indicated with the dotted line). Error bars indicate standard deviation between three independent experiments. * indicates siginificance *p* < 0.05. **b**. Quantitative luciferase reporter gene assays with the constructs containing 3xNF-kB response elements were performed in human HuH7 cells 48 h after indicated treatments and in total 72 h after the transfection with the indicated constructs. The bars represent the fold induction of luciferase activity normalized to the control state without any treatment (indicated with the dotted line). Error bars indicate standard deviation between three independent experiments. * indicates siginificance *p* < 0.05

Considering that PPARα-tr variant does not bind to PPREs and does not act in a dominant negative fashion on the full-length receptor, we hypothesized that PPARα-tr exerts its inhibitory functions via crosstalk to other direct regulators of these genes. In particular, *in silico* analyses of canonical elements using TRANSFAC database revealed presence of TCF/LEF binding regions within the promoters of proliferative genes used in this study. Thus, a series of transfections with the luciferase reporter constructs carrying TCF/LEF binding elements in combination with either PPARα-wt or PPARα-tr cDNAs were performed in HuH7 cells using co-stimulation of WNT/b-catenin pathway with the canonical natural WNT ligand, WNT3a. As shown in Fig. 6a, treatment with 20 ng/ml WNT3a significantly induced the luciferase signal more than ~ 5 fold compared to the solvent control. Interestingly, combination of WNT3a treatment together with WY14,643 resulted in significant reduction of luciferase signal down to ~ 3 fold. Furthermore, co-transfection with PPARα-tr further decreased whereas transfection with PPARα-wt did not have any effect on the TCF/LEF-mediated promoter activity. Finally, treatment with WNT3a alone in combination with PPARα-tr resulted in a similar downregulation of luciferase signal as in the presence of WY14,643.

Additionally we assessed activity of PPARa isoforms on the *nuclear factor* 'kappa-light-chain-enhancer' of activated B-cells, NF-kB – mediated regulation of the pro-inflammatory genes following treatment with IL-6. As shown in Fig. 6b, induction of an acute phase response resulted in the significant induction of NF-RE mediated luciferase signal more than ~ 4 fold compared to the solvent control. As expected, combination of IL-6 treatment together with PPARα-wt overexpression resulted in significant reduction of luciferase signal down to ~ 3 fold. However, co-transfection with PPARα-tr further significantly decreased NF-RE mediated promoter activity, indicating stronger inhibitory function of PPARα-tr in comparison to PPARα-wt. We therefore suggest that an intricate crosstalk of PPARα-tr with WNT/β-catenin and NF-kB pathways might be a potential mechanism of PPARα-tr inhibitory activity on the expression of proliferative and inflammatory genes respectively, which definitely warrants further detailed investigation.

### PPAR**α**-tr inhibits proliferation of mouse hepatocytes and human cancer cells

**Fig. 7:**
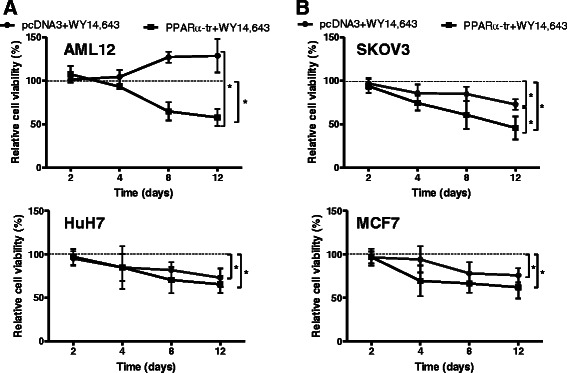
Cell viability analysis following PPARα-tr overexpression in mouse and human cancer cells. AML12 (**a**, top), HuH7 (**a**, bottom), SKOV3 (**b**, top) and MCF7 (**b**, bottom) cells were transfected with indicated constructs treated with 100 μM WY14,643 and cell viability was measured using CellTiterGlo® assay at the indicated days. The viability curves are shown relative to the pcDNA3-transfected cells treated with solvent control, DMSO, set as 100 % (dashed line). Error bars indicate standard deviation between three independent experiments measured in triplicates. * indicates significance *p* < 0.05

To further explore whether inhibitory effects of PPARα-tr on the expression of proliferative genes affects cell viability, AML12 cells were treated with WY14,643 in the presence or absence of PPARα-tr (Fig. 7a, upper panel). In line with previous results on the WY14,643-mediated inducibility of proliferative genes in mouse, increased proliferation of mouse hepatocytes towards treatment with WY14,643 was observed (Fig. 7a, upper panel, line with circles). Remarkably, overexpression of PPARα-tr in these cells not only reversed the effect of the PPARα activator, but further decreased viability on day 12 substantially below control levels (vector-transfected cells treated with WY14,643). In contrast, treatment of HuH7 cells with WY14,643 alone lead to decreased cell viability on day 12 (Fig. 7a, bottom panel, line with circles). However, transfection of PPARα-tr did not lower HuH7 cell viability (Fig. 7a, bottom panel, line with squares). To test whether viability of other cancer cells is susceptible towards fibrates and overexpression of PPARα-tr, we performed the same experiments in the human ovarian carcinoma cell line, SKOV3 (Fig. 7b, upper panel) and the human breast cancer cell line, MCF7 (Fig. 7b, bottom panel). In both cases, treatment with WY14,643 significantly reduced cell viability until day 12. Transfection of SKOV3 cells with the truncated variant of PPARa led to the further significant reduction of cell viability while the effect on MCF7 cells was also consistent over time, yet did not reach statistical significance (Fig. 7b, bottom panel).Of note, the cell viability curves generated for SKOV3 cells overexpressing either PPARα-wt or PPARα-tr cDNA in the presence of two additional PPARα ligands, clofibrate and GW7647, confirmed that the observed effects are not due to a ligand dependent effect on different splice variants (Additional file 4: Figure S3).

## Discussion

Despite its initial identification and characterization in the late 1990s, the function of the C-terminally truncated PPARα splice variant in human cells has remained speculative. Although *in vitro* data suggested a possible dominant negative function, no direct or supporting *in vivo* data had been provided so far. On the other hand, several recent papers suggested an involvement of the variant in the lack of fibrate-mediated hepatocarcinogenesis in humans, but data supporting this hypothesis are also lacking [4, 12, 21]. Here we used human liver samples to characterize expression and interindividual variability of the two PPARα splice variants in relation to genome-wide target gene expression, which indicated potentially differential roles. Assessment of the distribution of each PPARα form in the cohort of liver tissues revealed high correlation of mRNA levels of both variants to each other. This is in accordance with Hanselman et al. [14], who also reported high correlation of mRNA levels between PPARA-wt and PPARA-tr in 18 human livers (r_s_ = 0.75). However, our assessment of the protein isoforms in a cohort of human liver samples revealed the interesting novel observation, that the protein levels of the canonical and variant splice receptor forms are much less correlated than the transcripts, indicating significant posttranscriptional regulation. It should be interesting to investigate whether microRNAs contribute to this divergent expression.

Newly designed isoform-specific tools used to study their function in human and mouse cell models demonstrated that silencing of PPARα-tr in human hepatocytes had no effect on several classical PPARα target genes, thus arguing against a general function of the splice variant as dominant negative regulator. Instead, the variant isoform appears to have an autonomous function as negative regulator of proliferative and pro-inflammatory genes. As shown in Fig. 4b (upper panel), we did not observe any effect of specific siRNA-mediated inhibition of the endogenous PPARα-tr on the expression of ACOX1 or other canonical PPARα target genes in primary human hepatocytes of three independent donors. Although we cannot absolutely exclude any dominant-negative activity of PPARα-tr on the full-length PPARα-wt, a general function as such appears to be unlikely. This discrepancy between the earlier *in vitro* study [9] and our study could be due to several reasons. It is possible that the difference is merely of quantitative nature, i.e. the true effect may have been overestimated by the *in vitro* constructs used in the earlier study, and they may be too small to be detectable in our model system of primary human hepatocytes. Another explanation could be that the negative effects observed depend on nuclear translocation of PPARα-tr, as suggested previously. While in the former study nuclear translocation had been achieved by a nuclear localization signal fused to the N-terminus, translocation *in vivo* may depend on physiological conditions.

Our data strongly suggest that PPARα-tr acts independently of PPARα-wt in negatively regulating proliferative and pro-inflammatory gene sets. This conclusion is based on several complementary observations. First, bioinformatic analyses revealed that there were virtually no genes showing positive correlation to the canonical receptor and simultaneous negative correlation to the splice variant, as would be expected if there was a dominant negative influence. Second, our specifically designed gene silencing probes clearly showed strong effects on several proliferative and proinflammatory genes, whereas the knock-down of the canonical receptor had no effect. Third, essentially complementary results were obtained by specific overexpression of the two variants in various cells.

Evidence for an involvement of PPARα-tr in transcriptional regulation of specific genes distinct from PPARα-wt pathways has also been provided by others. Beaumont et al. described a mechanism of apoptosis induction following overexpression of PPARα-tr in human cardiomyocytes via downregulation of the anti-apoptotic protein, Bcl-2 [2]. Goikoetxea et al. found lower expression of PPARα-wt and higher expression of PPARα-tr (*P* < 0.001) in endomyocardial septal biopsies from patients with heart failure [11]. The apoptose index was directly correlated to PPARα-tr suggesting that PPARα-tr has a role in the pathophysiology of the left ventricle. Interestingly, divergent gene regulation between a canonical and a splice variant has also been found for the beta isoform splice variant of the human GR [15]. This truncated form may alter gene transcription independent from the canonical receptor and increased GR-beta levels were correlated with glucocorticoid resistance and the occurrence of several immune-related diseases. It is important to mention that the significant effects of PPARα-tr were observed only in the presence of PPARα ligand, WY14,643. Since PPARα-tr lacks the ligand-binding domain, both ligand-mediated activation of the full-length protein or PPARα-independent effects of WY14,643 could be involved in the activation of PPARα-tr. Indeed, it is known that PPARα regulates its own expression [27] and thus can be associated with the direct generation of its own alternative splice variant. Furthermore, regulation via post-translational modifications could be an additional mechanism of PPARα-tr activation [6, 41]. It is important to mention that our experiments using additional PPARα agonists, such as clofibrate or GW7647, further confirmed ligand-independent nature of the differential functions of PPARα isoforms (Additional file 4: Figure S3).

The interplay between PPARα and pro-inflammatory genes was studied previously [10, 20, 35]. Surprisingly, however, we could see no effect of siRNA-mediated downregulation of an endogenous PPARα-wt on the expression of pro-inflammatory genes in PHH (Fig. 4b, bottom panel). We speculate that anti-inflammatory PPARα-mediated effects can be attributed to the function of PPARα-tr via interference with NF-kB pathway. Indeed, luciferase reporter gene assays using luciferase constructs containing NF-kB response elements (NF-kB RE) further supported this hypothesis (Fig. 6b). Although overexpression of PPARα-wt resulted in the reduced NF-kB RE-mediated luciferase expression, transfection with PPARα-tr led to even stronger inhibition of the luciferase signal. Recently, we described a novel regulatory crosstalk between PPARα and WNT/β-catenin pathways [37]. Our study strongly indicates the intensive crosstalk between PPARα and β-catenin in the regulation of the downstream target genes of both transcription factors. Based on the luciferase reporter gene assays, we suggest that truncated PPARα variant might be involved in the negative regulation of proliferative gene expression presumably via interaction with β-catenin. Interestingly, WNT/β-catenin signalling pathway was also significantly enriched with genes negatively correlating with PPARα-tr protein expression in our bioinformatic analysis of liver samples (Fig. 3, right box), implying involvement of PPARα-tr in the regulation of other factors within this cascade. Undoubtedly, additional studies are required for the further understanding of the interplay between these two pathways.

Determining the mode of action of PPARα ligands in causing liver cancer in rodent models and the mechanism of the species differences are of great importance since fibrate drugs are widely used in the clinics. Furthermore, new generation drugs, with PPARα agonist activity (EC_50_) of more than 100-fold greater than the fibrates, are under development by the pharmaceutical industry [43]. It is meanwhile clear that fibrates itself do not cause genetic damage but rather metabolic alterations or interference with the cell cycle, resulting from sustained receptor activation contribute to oxidative stress induced DNA damage promoting hepatocarcinogenicity. Furthermore, several studies suggested induced expression of c-Myc protein to be the mechanism contributing to PPARα ligand-induced hepatocellular proliferation [30]. Our data showing the prevention of c-Myc induction by PPARα-tr following PPARα activation supports this hypothesis (Fig. 4b and Fig. 5a). Additionally, we observed higher susceptibility of several key proliferative genes to the WY14,643-mediated PPARα induction in mouse compared to human cells (Fig. 5a). Thus, we suggest that both higher induction of proliferative genes and lack of the inherent inhibitory control-mechanism by the truncated splice form contribute to fibrate-induced carcinogenesis in mice.

Several recent studies have revealed that PPARα ligands suppress the growth of human cancer lines, including colon, breast, endometrial and skin, in vitro [19, 31, 33]. Clofibric acid inhibits the growth of human ovarian cancer in mice [45]. Furthermore, it was shown that PPARα agonist, WY14,643, suppresses tumorigenesis in a PPARα-dependent manner [28]. The antitumor properties of PPARα ligands appear to be mediated primarily by their direct and indirect anti-angiogenic effects and their anti-inflammatory activity but also by direct antitumor effects, without so far clearly defined mechanisms. Based on our studies, we suggest that the truncated PPARα splice variant provides protective mechanism in acting as an endogenous inhibitor of proliferative and pro-inflammatory genes in human cells and its absence in mouse may explain species-specific differences in fibrate-induced hepatocarcinogenesis. We hope our findings will help in further development and improvement of anti-cancer therapy using already approved PPARα agonists.

## Conclusions

Based on our studies, we suggest that the truncated PPARα splice variant exerts antitumor effects via synergistic downregulation of proliferative and anti-inflammatory genes in human cells. Its absence in mouse may explain species-specific differences in fibrate-induced hepatocarcinogenesis.

## Methods

### Cell lines and treatments

*Primary human hepatocytes (PHH)* were isolated from partial liver resections by collagenase digestion as described previously [5]. Cells were cultured in William’s E Medium (Invitrogen Life Technologies, Darmstadt, Germany), supplemented with 10 % fetal bovine serum (FBS) (PAA Laboratories GmbH, Pasching, Austria), 1 % penicillin/streptomycin (GIBCO, Carlsbad, USA), 1 mM glutamine (GIBCO, Carlsbad, USA), 16 I.U. human insulin (Sanofi, Frankfurt, Germany), 0.1 % dimethyl sulfoxide (DMSO) (Sigma-Aldrich, Steinheim, Germany), and 50 mM dexamethasone (Sigma-Aldrich, Steinheim, Germany). Medium was changed daily.

Mouse *AML12* and human hepatoma *HuH7* cells were cultured at 37 °C with 5 % CO_2_ concentration and passaged every 3–4 days by the Trypsin/EDTA method. HuH7, SKOV3 and MCF7 cells were cultured in Dulbecco’s Modified Eagle Medium (DMEM) with 10 % fetal calf serum (FCS) gold (PAA Laboratories GmbH, Pasching, Austria), 1 % penicillin/streptomycin, and 1 % pyruvate. AML12 cells were cultivated in DMEM-F12 medium with 10 % FCS, 1 % penicillin/streptomycin, and 1 % glutamine.

For the induction experiments cells were treated for the indicated times either with 100 μM of WY14,643, 100 μM of clofibrate or 10 μM of GW7647 (all purchased at Sigma-Aldrich, Steinheim, Germany) dissolved in DMSO, or with 10 ng/μl of human recombinant interleukin-6 (PromoCell GmbH, Heidelberg, Germany) in phosphate buffered saline (PBS) (GIBCO, Carlsbad, USA), supplemented with 0.1 % bovine serum albumin (BSA) (Sigma-Aldrich, Steinheim, Germany), or vehicle only (DMSO or PBS + 0.1 % BSA). During the knock-down experiments, the chemicals were added 4 hours after the siRNA transfections. In the overexpression experiments, substances were added 24 h after the transfection of the vectors. The time on the diagrams indicates time upon start of the treatments.

### Human liver cohort

Liver tissues and corresponding blood samples were previously collected from 150 patients of Caucasian ethnicity (71 males and 79 females; average age of the subjects 58 ± 14 y). Patients who suffered from hepatitis, cirrhosis, or alcohol abuse were excluded. All tissue samples had been examined by a pathologist and only histologically non-tumorous tissue was used [17]. The study was approved by the ethics committees of the medical faculties of the Charité, Humboldt University, and of the University of Tuebingen and conducted in accordance with the Declaration of Helsinki. Written informed consent was obtained from each patient.

### Quantitative real-time RT-PCR analysis

Total RNA was isolated using the RNeasy Mini Kit, including on-column genomic DNA digestion with RNase free DNase Set (Qiagen, Hilden, Germany) as previously described [39]. RNA integrity and quantity were analyzed with the Agilent 2100 Bioanalyzer using the RNA 6000 Nano Kit (Agilent Technologies, Waldbronn, Germany). Synthesis of cDNA was performed with 500 ng RNA using Taqman Reverse Transcription Reagents (Applera GmbH, Darmstadt, Germany). Quantification of PPARα target gene expression was performed either using ABI Prism 7900HT Taqman (Applied Biosystems) or Fluidigm’s BioMark HD high-througphut quantitative chip platform (Fluidigm Corporation, San Francisco, USA), following the manufacturer’s instruction [34]. The following validated gene expression TaqMan® assays from Applied Biosystems were used: GAPDH (Hs00266705_g1), ACOX1 (Hs01074241_m1), APOC3 (Hs00163644_m1), HMGCS2 (Hs00985427_m1), PDK4 (Hs01037712_m1), MYC (Hs00153408_m1), CDK1 (Hs00938777_m1), CDK4 (Hs00262861_m1), PCNA (Hs00696862_m1), IL-1β (Hs01555410_m1), PTGS2 (Hs00153133_m1), CCL2 (Hs00234140_m1), TNF (Hs01113624_g1)Gapdh (Mm99999915_g1), c-Myc (Mm00487804_m1), Cdk1 (Mm00772472_m1), Cdk4 (Mm00726334_s1), Pcna (Mm00448100_g1), Il-1β (Mm00434228_m1), Ptgs2 (Mm00478374_m1), Ccl2 (Mm00441242_m1), Tnfα (Mm00443258_m1). The mRNA expression levels were normalized to glyceraldehyde-3-phsophate dehydrogenase (*GAPDH/Gapdh*) mRNA levels.

For the detection of PPARα isoform expression in the liver samples (*N* = 150), specific primers and the probe for the detection of only wild-type transcript, positioned within the 6^th^ exon, and for truncated variant, positioned on the 5^th^ and 7^th^ exon and the probe exactly on the junction site, were designed (for schematic representation see Fig. 1). Expression plasmids for each isoform were in parallel run in dilutions for the calibration of absolute amount of the transcripts in the liver samples as it was previously described [16] and normalized to RPLP0 (Hs99999902_m1) expression.

### Expression constructs for PPARα isoforms

Human PPARα expression plasmid pcDNA3-hPPARα was a kind gift of T. Tanaka (24). Expression vector of PPARα splice variant missing exon 6 was constructed from pcDNA3-hPPARα by amplification of fragment F1 using primers T7_fw (TAATACGACTCACTATAGGG) and ex5/7_rev (GTCACACAACGCCTTTTGTCATACATGATATGG) and fragment F2 using primers ex5/7_fw (TATGACAAAAGGCGTTGTGTGACATCCCG) and pcDNA_rev (TAGAAGGCACAGTCGACG). Using both fragments, a PCR fusion (sequence overlap is underlined) was performed using T7_fw and pcDNA_rev primers to form a 1429bp product, which was cloned into pCR4-TOPO vector (Life technologies, Carlsbad, Germany). The correct fusion and complete cDNA sequence was confirmed by sequencing of both strands. Exchange of a 1.32kB *Bam*HI/*Ap*aI fragment in the full length parent derivative resulted in pcDNA3-hPPARα-tr.

### Western blot analysis

For the simultaneous measurements of PPARα isoforms in the cohort of liver samples, 50 μg of tissue homogenate was electrophoretically separated on a 10 % SDS-Polyacrylamide gel and subsequently transferred to a nitrocellulose membrane using a Trans-blot semi-dry Fastblot 44 transfer chamber (Biometra, Goettingen, Germany). After blocking with 5 % skim milk in TBST membranes were incubated with the primary antibodies in 1 % skim milk solution in TBST. For the detection of the protein levels of each isoform we used a polyclonal antibody detecting both isoforms (rabbit anti-human PPARa, CAYMAN No. 101710, dilution 1:500). The following additional antibodies and dilutions were used: mouse anti-β-Actin (Sigma-Aldrich, A5441, 1:500) was used to detect β-Actin for normalisation; goat-anti-rabbit-IRD800 (Li-COR, 926–32214, 1:10.000) and goat-anti-mouse-IRD650 (Li-COR, 926–68074, 1:10.000) were fluorescently labeled secondary antibodies. Membranes were washed 4 times with TBST for 15 min before they were incubated with the secondary antibody for 30 min at room temperature. Detection was performed with a Li-COR Odyssey CLx fluorescence reader (Bad Homburg, Germany). Serial dilutions of a liver homogenate with good expression of both PPARα isoforms were run on each gel and used for inter- and intra-membrane calibration.

### Transfections with siRNAs and cDNAs

For the RNAi experiments, PHHs were transfected with 20 nM siRNAs using 10 pmol of Lipofectamine® RNAiMAX Transfection Reagent (Life Technologies, Carlsbad, Germany) in 12-well plates with serum free medium. The indicated siRNAs specifically targeting PPARα variants were custom designed and a non-targeting siRNA as a negative control (Lo GC Duplex #2) were obtained from Life Technologies (Carlsbad, Germany). To the cells containing 100 μl culture medium, 100 μl of the transfection cocktail was added to each well after 4–6 hours of incubation time following arrival. For overexpression, 200 ng of cDNA vectors were mixed with 2 μl of Lipofectamine® 3000 Reagent (Life Technologies, Carlsbad, Germany) and upon 20 minutes of complex formation, the liposomes were given to the cells plated in 24-well plates for the analysis of gene expression.

### Luciferase reporter gene assays

The reporter gene assays were performed as previously described [38]. The pGL3 containing TCF4/LEF response element upstream of luciferase genes was a gift of Prof. Wehkamp [18]. pGL4.32 Vector containing five copies of an NF-κB response element (NF-κB-RE) that drives transcription of the luciferase reporter gene was purchased by Promega (Mannheim, Germany).

### CellTiter Glo luminescent cell viability assay

The CellTiter-Glo luminescent cell viability assay (Promega, Mannheim, Germany) is based on the quantitation of ATP, reflecting the presence of metabolically active or viable cells. Cells were seeded in 96-well clear bottom opaque plates (6005199, Perkin Elmer, Rodgau Germany) at 1×10^3^ cells/well density. The cells were transfected with pcDNA3 control vector or pcDNA3, containing cDNA of PPARα-tr and cultured for 12 days. At day 1 after seeding, cells were treated with 20 ng/ml WNT3a (Sigma, Taufkirchen, Germany; dissolved in 0.1 % bovine serum albumin) and WNT3a treatment was repeated without additional medium change at days 5 and 6. Medium containing WY14,643 was changed daily to keep PPARα activated. On the indicated days, an equal volume of reconstituted CellTiter-Glo reagent was added to 50 μl of cultured cells in three wells per time point and treatment. The contents were mixed for 2 minutes to induce cell lysis. The luminescent signal was measured using multimode reader Enspire (Perkin Elmer, Rodgau, Germany).

### Statistical analyses

Statistical analyses were performed using software R-2.11.0 [3]. For the genome-wide correlation analyses, whole genome gene expression profiles of the 150 particularly well-characterized samples were generated by using Human-WG-6v2 Expression BeadChips (Illumina, Eindhoven, Netherlands) and are publically available [32]. After combining synonymous probe sets and removal of probes that did not correspond to a mapped gene, 24,754 genes were selected for further analyses. Spearman correlation was calculated between mRNA transcripts and the protein levels of PPARα-wt, PPARα-tr, and PPARα-wt/PPARα-tr ratio in 150 liver samples. Transcripts correlated with each PPARα protein form at *r*_*s*_ ≥ 0.3 and *p* < 0.01 were used for the enrichment analysis using the pathway database, Reactome (www.reactome.org).

For demonstration of gene expression changes, the mean fold changes as obtained from the ΔΔCT-method and their standard deviations were calculated. To determine the significance of gene expression changes, grouped t-test with Bonferroni post-hoc-test for multiple comparisons was applied using GraphPad Prism 5.0.4 software (GraphPad Software, Inc., La Jolla, USA).

## Additional files

Additional file 1: Table S1. The 20 highest ranked genes in the defined groups according to Spearman correlation analysis of genome-wide gene expression data (see also Fig. 3).
Additional file 2: Figure S2. Expression levels of the target genes following transfections with siRNAs in the absence of WY14,643 (A, B) or IL-6 (C). A. PHHs (*n* = 3) were transfected with siRNAs targeting PPARA-wt transcript only (siRNA-wt), PPARA-tr only (siRNA-tr), or both transcripts (siRNA-tot). Total and specific mRNA levels were determined by using specific TaqMan assays in comparison to non-targeting siRNA (siRNA-ctr; set to 1 and shown in white bars). Results represent means of three PHH donors with two individual replicates. B. qRT-PCR analysis of the selected proliferative genes following overexpression of each PPARα isoform and treatment with the solvent control, DMSO, of mouse AML12 (top) and human hepatoma HuH7 cells (bottom). C. Quantitative qRT-PCR analysis of the selected pro-inflammatory genes following overexpression of each PPARα isoform and treatment with the solvent control, PBS, of mouse AML12 (top) and human hepatoma HuH7 cells (bottom).
Additional file 3: Figure S1. Analysis of endogenous expression of PPARα-wt and PPARα-tr in mouse and human cell lines. A. Quantitative real-time RT-PCR analysis of PPARA-wt (black bars) and PPARα-tA (grey bars) mRNA levels in selected liver homogenate (LH), primary human hepatocytes (PHH), mouse AML12 (AML12) and human Huh7 cells. The data are represented relative to the liver homogenate (LH) (set as 1). B. Representative Western blot analysis of PPARα-wt and PPARα-tr protein expression in the liver homogenate (LH), AML12 and PHH cells. C. Despite of PPARA-tr mRNA expression, we could not detect PPARα-tr protein expression in HuH7 cells (the most right lane at the western blot). To exclude technical problems, western blot analysis was performed following transfection of HuH7 cells with PPARα-tr expression plasmid (left lane on the blot) and 24 h later with indicated siRNAs. Densitometric evaluation of the bands is shown on the bottom and given relative to the PPARα-tr overexpression conditions.
Additional file 4: Figure S3. Cell viability analysis following PPARα-tr overexpression in human ovarian cancer SKOV3 cells. SKOV3 cells were transfected with indicated constructs treated with 100 μM clofibrate (A) or 10 μM GW7647 (B) and cell viability was measured using CellTiterGlo® assay at the indicated days. The viability curves are shown relative to the pcDNA3-transfected cells treated with solvent control, DMSO, set as 100 % (dashed line). Error bars indicate standard deviation between three independent experiments measured in triplicates. * indicates significance *p* < 0.05.

## Abbreviations

ACOX1: Acyl-CoA oxidase 1
APOC3: Apolipoprotein C-III
CCL2: Chemokine (C-C motif) ligand 2
CDK1: Cyclin-dependent kinase 1
CDK4: Cyclin-dependent kinase 1
DMSO: Dimethylsulfoxide
HMGCS2: 3-hydroxy-3-methylglutaryl-CoA synthase 2
IL1B: Interleukin 1 beta
MYC: V-Myc Avian Myelocytomatosis Viral Oncogene Homolog
PCNA: Proliferating cell nuclear antigen
PDK4: Pyruvate dehydrogenase kinase, isozyme 4
PHH: Primary human hepatocytes
PPARα: Peroxisome proliferator activated receptor alpha
PTGS2: Prostaglandin-endoperoxide synthase 2
siRNA: small interfering RNA
TNFα: Tumor necrosis factor alpha
WY14,643: 4-chloro-6-(2,3-xylidino)-2-pyrimidinylthio acetic acid
