# Supplementary material for: The truncated splice variant of peroxisome proliferator-activated receptor alpha, PPARα-tr, autonomously regulates proliferative and pro-inflammatory genes
Source: BMC Cancer. 2015 Jun 30;15:488. doi: 10.1186/s12885-015-1500-x (PMC4485637; doi:10.1186/s12885-015-1500-x)
Supplement: Additional file 1: Table S1. — The 20 highest ranked genes in the defined groups according to Spearman correlation analysis of genome-wide gene expression data (see also Fig. 3). [file 12885_2015_1500_MOESM1_ESM.docx]

**Supplementary Table S1:** The 20 highest ranked genes in the defined groups according to Spearman correlation analysis of genome-wide gene expression data (see also Fig.3).

| **Gene** | **Spearman correlation (rs)** | **P value** | **Gene full name (acc. to HUGO Gene Nomenclature Committee)** |
| --- | --- | --- | --- |
| **Group A: Top 20 genes positively correlating with PPARα-wt** | | | |
| **SLC39A5** | 0.56 | 3.0707E-09 | Solute Carrier Family 39 (Zinc Transporter), Member 5 |
| **ALDH9A1** | 0.55 | 5.18684E-09 | Aldehyde Dehydrogenase 9 Family, Member A1 |
| **FGF6** | 0.55 | 5.53811E-09 | Fibroblast Growth Factor 6 |
| **ALAD** | 0.55 | 6.65438E-09 | Aminolevulinate Dehydratase |
| **CMBL** | 0.55 | 1.08669E-08 | Carboxymethylenebutenolidase Homolog (Pseudomonas) |
| **LGTN** | 0.54 | 2.04645E-08 | Ligatin |
| **FXYD1** | 0.53 | 3.1897E-08 | FXYD Domain Containing Ion Transport Regulator 1 |
| **NR2F6** | 0.53 | 3.53663E-08 | Nuclear Receptor Subfamily 2, Group F, Member 6 |
| **GALT** | 0.52 | 7.45464E-08 | Galactose-1-Phosphate Uridylyltransferase |
| **EBP** | 0.52 | 7.47838E-08 | Emopamil Binding Protein (Sterol Isomerase) |
| **MRPS24** | 0.52 | 9.96806E-08 | Mitochondrial Ribosomal Protein S24 |
| **SC5DL** | 0.52 | 1.05143E-07 | Sterol-C5-Desaturase |
| **POFUT1** | 0.52 | 1.15361E-07 | Protein O-Fucosyltransferase 1 |
| **ASMTL** | 0.51 | 1.30616E-07 | Acetylserotonin O-Methyltransferase-Like |
| **MAMDC4** | 0.51 | 1.31734E-07 | MAM Domain Containing 4 |
| **TRAPPC6A** | 0.51 | 1.81798E-07 | Trafficking Protein Particle Complex 6A |
| **IL11RA** | 0.51 | 2.36586E-07 | Interleukin 11 Receptor, Alpha |
| **MADD** | 0.51 | 2.70384E-07 | MAP-Kinase Activating Death Domain |
| **PKLR** | 0.50 | 2.88881E-07 | Pyruvate Kinase, Liver And RBC |
| **TMEM97** | 0.50 | 2.98873E-07 | Transmembrane Protein 97 |
| **Group B: Top 20 genes positively correlating with PPARα-wt/PPARα-tr ratio** | | | |
| **HYDIN** | 0.42 | 5.14578E-05 | HYDIN, axonemal central pair apparatus protein |
| **CCDC140** | 0.42 | 7.54664E-05 | coiled-coil domain containing 140 |
| **SUPT3H** | 0.41 | 0.000134531 | suppressor of Ty 3 homolog (S. cerevisiae) |
| **RBBP6** | 0.40 | 0.000190981 | retinoblastoma binding protein 6 |
| **MARVELD1** | 0.39 | 0.000331092 | MARVEL domain containing 1 |
| **TESSP5** | 0.39 | 0.000340592 | protease, serine, 45 |
| **CREB5** | 0.39 | 0.000359863 | cAMP responsive element binding protein 5 |
| **FGF6** | 0.38 | 0.000614873 | fibroblast growth factor 6 |
| **ZNF672** | 0.37 | 0.000754042 | zinc finger protein 672 |
| **DLG5** | 0.37 | 0.000765304 | discs, large homolog 5 (Drosophila) |
| **OR2T11** | 0.37 | 0.000777757 | olfactory receptor, family 2, subfamily T, member 11 (gene/pseudogene) |
| **TPD52L3** | 0.36 | 0.001074641 | tumor protein D52-like 3 |
| **MLLT6** | 0.36 | 0.001153016 | myeloid/lymphoid or mixed-lineage leukemia (trithorax homolog, Drosophila); translocated to, 6 |
| **RGS16** | 0.36 | 0.001216584 | regulator of G-protein signaling 16 |
| **CHRNA2** | 0.36 | 0.001229617 | cholinergic receptor, nicotinic, alpha 2 (neuronal) |
| **GP1BA** | 0.36 | 0.001235393 | glycoprotein Ib (platelet), alpha polypeptide |
| **PLA2G4E** | 0.36 | 0.001322445 | phospholipase A2, group IVE |
| **PCOLCE** | 0.36 | 0.001333638 | procollagen C-endopeptidase enhancer |
| **MCL1** | 0.36 | 0.001402538 | myeloid cell leukemia 1 |
| **ID2B** | 0.36 | 0.001496495 | inhibitor of DNA binding 2B, dominant negative helix-loop-helix protein (pseudogene) |
| **Group C: Top 20 genes negatively correlating with PPARα-tr** | | | |
| **DUXAP3** | -0.45 | 1.12242E-05 | double homeobox A pseudogene 3 |
| **KLC2** | -0.43 | 3.80467E-05 | kinesin light chain 2 |
| **SPOCD1** | -0.42 | 6.87395E-05 | SPOC domain containing 1 |
| **PTPN7** | -0.42 | 7.58158E-05 | protein tyrosine phosphatase, non-receptor type 7 |
| **CTRL** | -0.41 | 8.67704E-05 | chymotrypsin-like |
| **NCF4** | -0.41 | 9.79837E-05 | neutrophil cytosolic factor 4, 40kDa |
| **PTGS2** | -0.41 | 0.003267197 | prostaglandin-endoperoxide synthase 2 (prostaglandin G/H synthase and cyclooxygenase) |
| **KCNQ2** | -0.41 | 0.000135235 | potassium voltage-gated channel, KQT-like subfamily, member 2 |
| **VSTM1** | -0.41 | 0.000147137 | V-set and transmembrane domain containing 1 |
| **EMR1** | -0.39 | 0.000295094 | egf-like module containing, mucin-like, hormone receptor-like 1 |
| **CDK1** | -0.39 | 0.000330404 | cyclin-dependent kinase 1 |
| **ITGA2B** | -0.39 | 0.000385397 | integrin, alpha 2b (platelet glycoprotein IIb of IIb/IIIa complex, antigen CD41) |
| **CST7** | -0.39 | 0.000399134 | cystatin F (leukocystatin) |
| **NCDN** | -0.38 | 0.000532576 | neurochondrin |
| **SNX32** | -0.38 | 0.000535282 | sorting nexin 32 |
| **KIR2DL4** | -0.38 | 0.000547067 | killer cell immunoglobulin-like receptor, two domains, long cytoplasmic tail, 4 |
| **STK4** | -0.38 | 0.00056812 | serine/threonine kinase 4 |
| **ADM2** | -0.38 | 0.000651102 | adrenomedullin 2 |
| **CDK4** | -0.37 | 0.000889432 | cyclin-dependent kinase 4 |
| **H2AFB1** | -0.37 | 0.000950773 | H2A histone family, member B1 |
| **EP400NL** | -0.36 | 0.001234305 | EP400 N-terminal like |
| **LACTB** | -0.36 | 0.001251131 | lactamase, beta |
| **DCDC2** | -0.36 | 0.001285015 | doublecortin domain containing 2 |
