# Supplementary figures and images for: The truncated splice variant of peroxisome proliferator-activated receptor alpha, PPARα-tr, autonomously regulates proliferative and pro-inflammatory genes
Source: BMC Cancer. 2015 Jun 30;15:488. doi: 10.1186/s12885-015-1500-x (PMC4485637; doi:10.1186/s12885-015-1500-x)

# Supplementary Figure S2

**A**

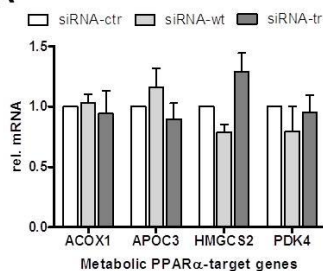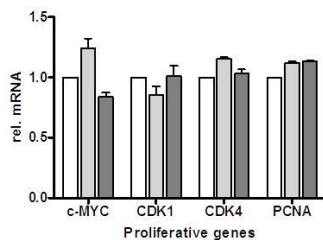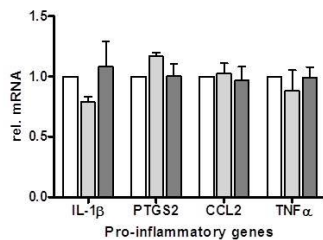

**B**

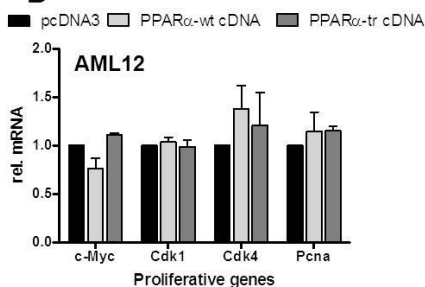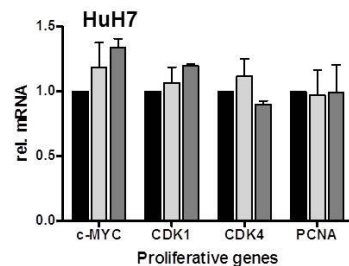

**C**

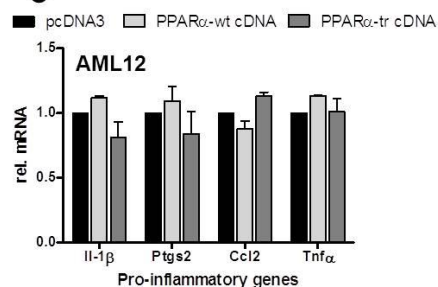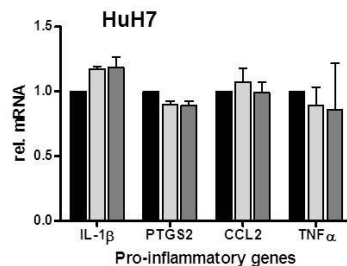

Supplement: Additional file 2: Figure S2. — Expression levels of the target genes following transfections with siRNAs in the absence of WY14,643 (A, B) or IL-6 (C). A. PHHs (n = 3) were transfected with siRNAs targeting PPARA-wt transcript only (siRNA-wt), PPARA-tr only (siRNA-tr), or both transcripts (siRNA-tot). Total and specific mRNA levels were determined by using specific TaqMan assays in comparison to non-targeting siRNA (siRNA-ctr; set to 1 and shown in white bars). Results represent means of three PHH donors with two individual replicates. B. qRT-PCR analysis of the selected proliferative genes following overexpression of each PPARα isoform and treatment with the solvent control, DMSO, of mouse AML12 (top) and human hepatoma HuH7 cells (bottom). C. Quantitative qRT-PCR analysis of the selected pro-inflammatory genes following overexpression of each PPARα isoform and treatment with the solvent control, PBS, of mouse AML12 (top) and human hepatoma HuH7 cells (bottom). [file 12885_2015_1500_MOESM2_ESM.pdf]

# Supplementary Figure S1

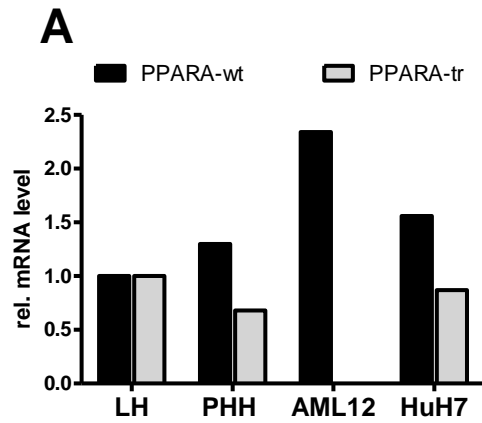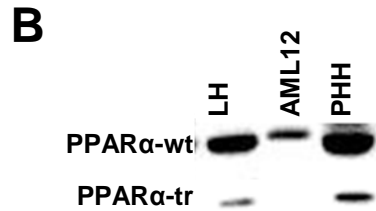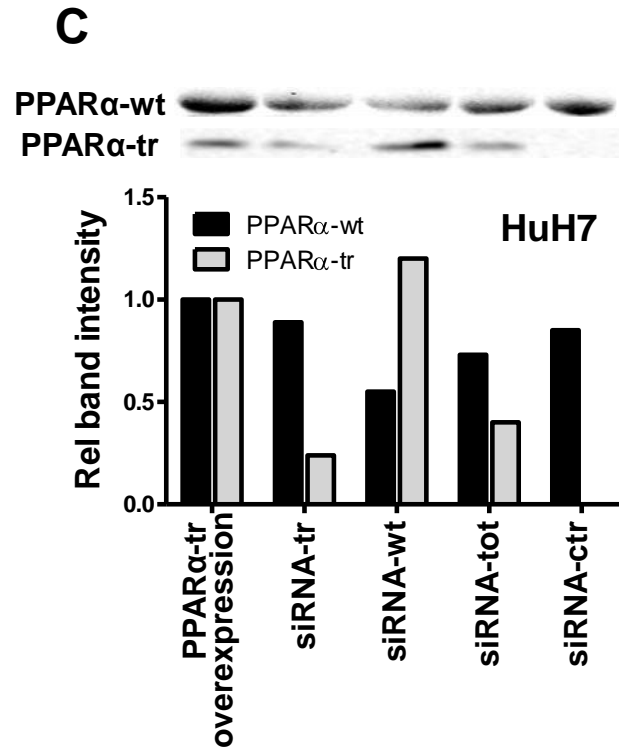

Supplement: Additional file 3: Figure S1. — Analysis of endogenous expression of PPARα-wt and PPARα-tr in mouse and human cell lines. A. Quantitative real-time RT-PCR analysis of PPARA-wt (black bars) and PPARα-tA (grey bars) mRNA levels in selected liver homogenate (LH), primary human hepatocytes (PHH), mouse AML12 (AML12) and human Huh7 cells. The data are represented relative to the liver homogenate (LH) (set as 1). B. Representative Western blot analysis of PPARα-wt and PPARα-tr protein expression in the liver homogenate (LH), AML12 and PHH cells. C. Despite of PPARA-tr mRNA expression, we could not detect PPARα-tr protein expression in HuH7 cells (the most right lane at the western blot). To exclude technical problems, western blot analysis was performed following transfection of HuH7 cells with PPARα-tr expression plasmid (left lane on the blot) and 24 h later with indicated siRNAs. Densitometric evaluation of the bands is shown on the bottom and given relative to the PPARα-tr overexpression conditions. [file 12885_2015_1500_MOESM3_ESM.pdf]

## Supplementary Figure S3

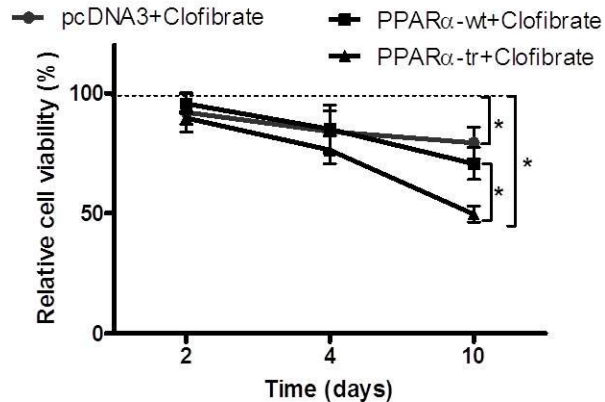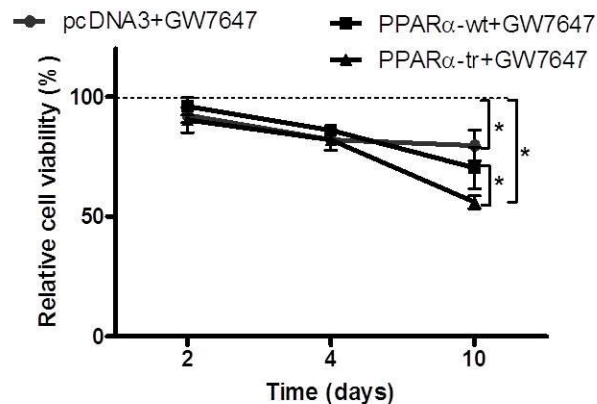

Supplement: Additional file 4: Figure S3. — Cell viability analysis following PPARα-tr overexpression in human ovarian cancer SKOV3 cells. SKOV3 cells were transfected with indicated constructs treated with 100 μM clofibrate (A) or 10 μM GW7647 (B) and cell viability was measured using CellTiterGlo® assay at the indicated days. The viability curves are shown relative to the pcDNA3-transfected cells treated with solvent control, DMSO, set as 100 % (dashed line). Error bars indicate standard deviation between three independent experiments measured in triplicates. * indicates significance p < 0.05. [file 12885_2015_1500_MOESM4_ESM.pdf]
